# A case report of response to crizotinib in chemotherapy-refractory metastatic gallbladder cancer with met amplification and acquired resistance resulting from the loss of MET amplification

**DOI:** 10.1093/pcmedi/pbab017

**Published:** 2021-07-30

**Authors:** Hongna Sun, Xiaofen Li, Shuang Dai, Xudong Shen, Meng Qiu

**Affiliations:** Department of Abdominal Cancer, Cancer Center, West China Hospital, Sichuan University, Chengdu 610041, China; Department of Abdominal Cancer, Cancer Center, West China Hospital, Sichuan University, Chengdu 610041, China; Department of Medical Oncology, Lung cancer center, West China Hospital, Sichuan University, Chengdu 611135, China; The Medical Department, 3D Medicines Inc., Shanghai 201202, China; Department of Abdominal Cancer, Cancer Center, West China Hospital, Sichuan University, Chengdu 610041, China

**Keywords:** metastatic gallbladder cancer, *MET* amplification, targeted therapy, ctDNA, acquired resistance; case report

## Abstract

Gallbladder cancer (GBC) is a highly invasive disease and the most prevalent malignancy of the biliary system. Patients with GBC are commonly diagnosed at a late stage and have an unfavorable prognosis. Palliative chemotherapy has been the standard care for recurrent or metastatic disease in the past decades. Recently, several targeted therapies have been investigated in advanced biliary tract cancer (BTC) including inhibitors of genes or pathways such as *FGFR2* fusions or rearrangements, *IDH1* mutations, and *NTRK* gene fusions. Also, several clinical studies involving molecular stratification have been performed in defined patient groups, for example, *BRAF V600E* and *HER2*. Mesenchymal epithelial transition*(MET)*encodes a tyrosine kinase receptor and its ligand hepatocyte growth factor is a proto-oncogene. Targeting the *MET* signaling pathway is an effective strategy in numerous cancer types. However, the poor efficacy of *MET* inhibitors has been demonstrated in several phase II studies, but currently no reports have explained the potential mechanisms of resistance to *MET* inhibitors in BTC. In this article, we report a case of metastatic GBC with *MET* amplification that exhibited a rapid response to crizotinib after the failure of two lines of chemotherapy. After the patient had progressed and discontinued crizotinib, cabozantinib was introduced. Analysis of circulating tumor DNA (ctDNA) by next-generation sequencing (NGS) indicated a loss of *MET* amplification status. To our knowledge, this is the first case study demonstrating the use of NGS in ctDNA to monitor the development of acquired resistance during anti-*MET* treatment in GBC.

## Introduction

Gallbladder carcinomas (GBCs) are the most prevalent malignancies in the biliary tract and are highly invasive diseases with late diagnosis and an unfavorable prognosis. The five-year survival rate of GBC is 5% for patients with stage III disease and 1% for patients with stage IV disease.^1^ Radical surgery remains the standard treatment for patients with locally resectable tumors. Palliative chemotherapy is the standard of care for patients with recurrent or metastatic disease; however, outcomes remain poor, with a median overall survival (OS) of <12 months. The poor outcomes in GBC are mainly due to low responses to first-line chemotherapy and the absence of effective second- or third-line therapeutic strategies. Recently, novel targeted therapies have been developed for the management of advanced biliary tract cancer (BTC) that have used next-generation sequencing (NGS) technology for patient stratification. Several targeted agents have been approved for advanced BTC by the FDA (Food and Drug Administration) including inhibitors of particular genes or pathways such as *FGFR2* fusions or rearrangements, *IDH1*mutations, *NTRK* gene fusions and MSI-H status. These alterations are present in around 0.1%–20% of patients with BTC and are less frequent in patients with GBC.^2,3^ Several precision medicine trials are ongoing, for example, *BRAF V600E* and *HER2*. These data suggest that it is very necessary to carry out accurate treatment for BTC based on molecular stratification.

Mesenchymal epithelial transition*(MET)*encodes a receptor tyrosine kinase and that is activated by binding of its ligand, hepatocyte growth factor (HGF).^4^ Dysregulation of the *MET* pathway involves amplification, fusion, overexpression, and mutation.^5^ Several studies have indicated *MET* overexpression in 11%–43% of intrahepatic cholangiocarcinoma (IHCC) and 16%–80% in extrahepatic cholangiocarcinoma (EHCC). *MET* amplification has been reported with a fairly low frequency ranging from 0% to 7% in IHCC and EHCC, and from 0% to 18% in GBC.^6^ Activation of *MET* signaling is involved in tumor angiogenesis, metastatic progression, and acquired resistance to anti-*EGFR* treatment.^7,8^ In a study by Hida and colleagues^,9^ expression of *MET* was also strongly correlated with tumor location, T category, AJCC stage, and perineural invasion. These factors can lead to successive local recurrence and poor prognosis in extrahepatic BTC. Therefore, targeting the *MET* signaling pathway is a potential treatment strategy. Combinations of multiple kinases or selective tyrosine kinase *MET* inhibitors (TKIs) and anti-*MET* antibodies, antibody–drug conjugates have been studied. For example, selective *MET* TKIs including capmatinib^10^ and the multikinase *MET* inhibitor crizotinib^11^ have shown promising efficacy in lung cancer patients with *MET* amplification and *MET* exon 14-alterations. However, the effectiveness and safety of *MET* inhibitors in GBC with *MET* amplification or mutations remains unknown.

Here, we report a case of terminal GBC with *MET* amplification that had metastasized to the lymph nodes and liver. The patient showed a rapid response to crizotinib after the failure of two lines of chemotherapy. Moreover, the *MET* amplification status was negative after the failure of anti-*MET* therapy.

## Case presentation

**Figure 1.**
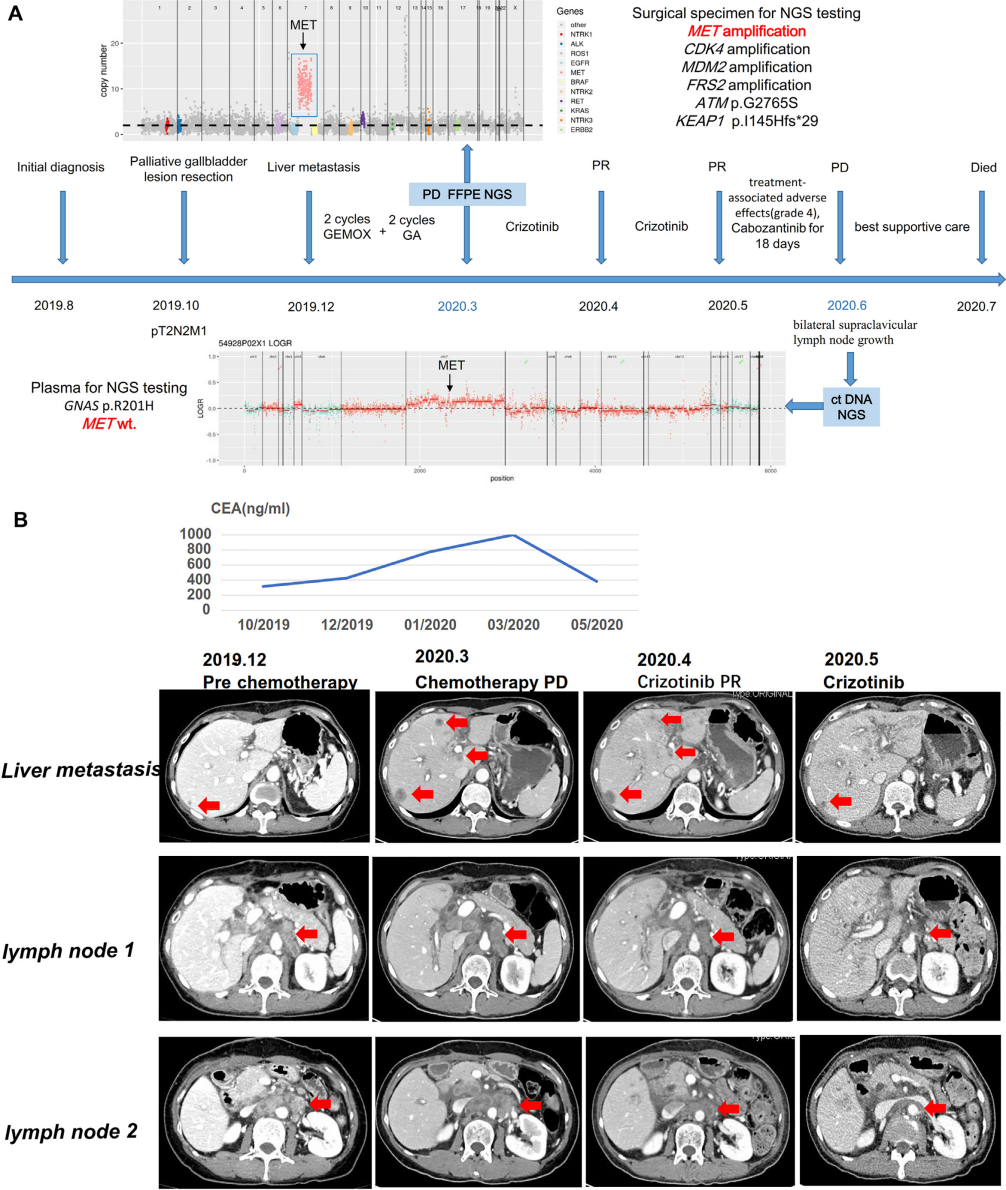
A. Timeline of treatment management and genetic changes in *MET* amplification of the patient with metastatic GBC. B. The levels of the tumor marker CEA and representative computed tomography (CT) images of tumor burden before chemotherapy and after two lines of chemotherapy and before crizotinib. Partial response to crizotinib was observed one month later with a continued partial response to crizotinib observed for 2 months. The figures include two lesions of the retroperitoneal lymph nodes and liver metastases. Abbreviations: CEA, carcinoembryonic antigen; FFPE, formalin-fixed and paraffin-embedded; ctDNA, circulating tumor DNA; NGS, next-generation sequencing; *MET*, mesenchymal epithelial transition; CDK4, cyclin dependent kinase 4; MDM2, murine double minute 2; FRS2, recombinant fibroblast growth factor receptor substrate 2; PD, progression disease; PR, partial response.

In August 2019, a 55-year-old woman visited our clinic with mild right upper abdominal pain on a numerical rating scale (NRS) of 2–3 with a poor appetite and weight loss (Fig. 1A). Magnetic resonance cholangiopancreatography of the abdomen discovered gallbladder lesions, retroperitoneal lymphadenopathy, and enlarged intraperitoneal lymph nodes. The serum CA19-9 level was 555 U/ml (normal range 0–22 U/ml) and the carcinoembryonic antigen (CEA) level was 316 ng/ml (normal ange, 0–3 ng/ml). The patient had no history of chronic diseases. On October 29, 2019, the patient underwent palliative resection of a gallbladder lesion and intraperitoneal lymph node biopsy. Histologically, the gallbladder lesion was diagnosed as poorly differentiated adenocarcinoma that had invaded the outer membrane and the lymph nodes were metastatic. The patient was diagnosed with GBC at pT2N2M1 and stage IVB.

One month after surgery, the patient had a new palpable hard nodule in the left neck that was 2 cm in diameter and had back pain. An enhanced abdomen computed tomography (CT) scan displayed a new nodule in the liver and the level of serum CA19-9 increased to over 1000 U/ml. CEA levels had also increased (Fig. 1B). The performance status (PS) score of the patient was 1. The patient was administered 2 cycles of GEMOX chemotherapy (gemcitabine plus oxaliplatin) as a first-line treatment on December 21, 2019. The patient then received 2 cycles of GA (gemcitabine plus paclitaxel-albumin) as a second-line treatment. Unfortunately, the best response to both regimens was progression disease according to the Response Evaluation Criteria in Solid Tumors version 1.1. The patient experienced intensified back pain to NRS 7–8. Fentanyl transdermal patches were used every 72 h and the PS score decreased to 2. CT evaluation after two lines of chemotherapy (pre-crizotinib) is shown in (Fig. 1B).

Genetic variations in the operative tumor specimens were assessed using NGS technology with a 733-gene panel (3D medicines Inc.) performed in a CLIA and CAP-certificated laboratory. Amplification of four genes*(MET, CDK4, MDM2, and FRS2)*was identified and the copy numbers were 5, 7, 5, and 6, respectively. NGS also revealed that the tumor harbored two mutations,*ATM*p.G2765S Exon57 and *KEAP1* p.I145Hfs*29 Exon2. After obtaining informed consent for the use of off-label targeted therapy, the patient started treatment with crizotinib at an initial dose of 250 mg q.d. in March 2020. After 2 months of treatment, the back pain was significantly reduced (NRS 2–3) and the left supraclavicular lymph node had almost disappeared. The serum CA19-9 level was significantly reduced to 383 U/ml. A partial response (PR) was observed by abdominal CT scan on reexamination with a significant reduction in the mass located in the liver as well as the retroperitoneal lymph node (Fig. 1B). However, the patient did not choose the recommended dose (250 mg b.i.d.) and finally discontinued crizotinib due to intolerable treatment-associated adverse effects including nausea (grade 4) and vomiting (grade 4) despite being prescribed antivomiting drugs. Supportive treatment was then given. A CT scan was performed and showed no evidence of disease progression.

Unfortunately, the patient developed rapid jaundice 10 days after discontinuation of crizotinib and underwent percutaneous transhepatic catheter drainage. One week later, bilirubin levels dropped and due to adverse effects another anti-*MET* targeted-drug (cabozantinib) was precribed. Cabozantinib was administered only for 18 days because of disease progression with bilateral supraclavicular lymph node growth. To reevaluate the dynamic change of the genetic characteristics, a peripheral blood sample was assessed for circulating tumor deoxyribonucleic acid (ctDNA) using NGS with an array of 61 genes (3D medicines Inc.) as the patient could not tolerate tissue biopsy. The ctDNA analysis was negative for *MET* amplification, and mutation of gene *GNAS* p.R201HExon8. The condition of the patient (PS 3–4) rapidly deteriorated and the best supportive care was given. The patient passed away on July 24, 2020. The OS of the patient was 9 months.

## Discussion

In this case study, the patient was treated with crizotinib after the failure of two lines of chemotherapy. NGS genomic profiling of the operative tumor specimen revealed *MET* amplification that confirmed a rapid PR within 2 months. The patient rapidly progressed with cabozantinib treatment potentially due to tumor heterogeneity. After the patient had progressed, crizotinib was discontinued and cabozantinib was introduced. ctDNA analysis was performed by NGS and showed that *MET* amplification was lost. In this case, it was indicated that *MET* inhibitors might be effective in harboring *MET* amplification advanced GBC offering a potential targeted therapy option in patients with GBC carrying this lesion. Also, the loss of *MET* amplification may be a mechanism that can promote resistance to subsequent anti-*MET* therapy, although the exact mechanism remains unclear. Mutation profiling of the patient's ctDNA by NGS revealed posttreatment dynamic changes in genomic status and may be used as a novel clinical strategy for personalized therapy in refractory BTC.

Traditionally, systemic chemotherapy with cisplatin plus gemcitabine has been the standard of care for patients with terminal BTC. Recently, targeted therapies have been proposed in the treatment of metastatic BTC. Several targeted agents have been approved by the FDA for advanced BTC including inhibitors of special genes or pathways such as *FGFR2* fusions or rearrangements, *IDH1*mutations, *HER2* amplifications and *NTRK* gene fusions. Precision therapies in BTC are still being explored. Targeted therapy, particularly agents targeting angiogenesis, have provided encouraging results, for example, regorafenib has been approved for advanced BTC by the FDA.

*MET* is an attractive new target that is involved in the pathogenesis of some malignancies. In recent years, *MET* inhibitors have been increasingly investigated and evaluated for the treatment of advanced BTC. Crizotinib is a small molecule tyrosine kinase inhibitor that inhibits *ALK*^12^, *ROS1*,^13^ and *MET*.^11^ IHCC patients harboring *EHBP1*–*MET* fusions present a continuous partial response for 8 months with crizotinib.^14^ The first phase II clinical trial of cabozantinib in terminal BTC patients using cabozantinib as a second- or third-line treatment demonstrated limited clinical efficacy in advanced refractory BTC with a median OS of 5.2 months and a median progression-free survival of 1.8 months.^15^ The *MET* inhibitor, tivantinib, has been combined with gemcitabine in BTC patients in a phase I study and patients demonstrated a partial response at 46% and stable disease at 27%.^16^ A randomized phase 2 study of merestinib is being conducted in patients with terminal BTC (NCT02711553).

Crizotinib has shown promise as a targeted therapy in lung cancers with *MET* amplification, yet the development of acquired resistance remains a major problem. Mechanisms of resistance to targeted drugs have been investigated in patients who initially benefited from *MET* inhibitors; for example, those with bypass signaling and second-site mutations, *MET* kinase domain mutations, and *MET* amplifications.^17^ However, the mechanism of drug resistance to *MET* inhibitors in advanced BTC patients with *MET* amplification remains unknown. In this case, the patient progressed after being treated with *MET* inhibitors and ctDNA analysis by NGS showed that *MET* amplification status was lost. Similarly, loss of *MET* amplification has been reported in some lung cancer patients who received first- or third-generation *EGFR*-TKI and crizotinib at progression.^18^ We hypothesize that loss of *MET* amplification may contribute to the occurrence of drug resistance and disease recurrence.

ctDNA is composed of short double-stranded DNA fragments that are released into the bloodstream by tumor cells during apoptosis or necrosis. ctDNA samples can be noninvasively and conveniently obtained in real-time during the disease and can be used to detect the genetic information of the tumor. This information can be used to predict treatment responses, monitor disease relapse, and track mechanisms of therapeutic resistance.^19^ Previous studies have shown that positive results on ctDNA NGS are highly consistent with NGS analysis of tissues in oncogenic driver alterations. Furthermore, in the study of Ikeda et al.,^20^ ctDNA NGS showed higher rates of *MET* alterations (including *MET* amplification) than the tissue NGS testing. ctDNA can avoid temporal and spatial (intratumor and intertumor) heterogeneity caused by tissue biopsy. Also, ctDNA may overcome the limitations of NGS in tissue biopsies including insufficient quantities of tissue and risks associated with repeated invasive procedures.^21^ Liquid biopsy provides the possibility to monitor genetic changes during crizotinib treatment of terminal GBC. A similar approach has been reported in non-small cell lung, colorectal, and breast cancer. These data indicate that dynamic monitoring of ctDNA is useful in selecting the appropriate therapeutic strategies to extend patient survival.

In addition to detecting *MET* amplification of GBC, there was *ATM* mutation of this patient. *ATM* mutation has been reported by Zhang et al. as a good target by olaparib in GBC,^22^ there may be a change of response by combining PARP inhibitors. However, this patient was in poor health and she discontinued crizotinib due to intolerable treatment-associated adverse effects despite being prescribed the relevant drugs. During the late stage of disease development, the patient could not tolerate anticancer therapy as the physical status score was 3–4. Nevertheless, this is a good choice for patients of good health; meanwhile, this combined treatment strategy deserves to be explored for its efficacy and safety in subsequent studies. This patient was treated with a reduced dose of crizotinib due to poor health status. A similar case has been reported in the study of Yang et al.,^13^ where an elderly female patient harboring *ROS1* rearrangement non-small cell lung cancer achieved a stable disease for 14 months on a reduced dose of crizotinib treatment.

This report has several limitations. Tissue was prioritized for genetic profiling after disease progression. We used peripheral blood samples to assess ctDNA using NGS because the patient could not tolerate tissue biopsy. However, ctDNA is the best alternative for when a conventional biopsy is unavailable or when insufficient quantities of DNA are available for sequencing. Also, the difference in the panel size of the genes in two tests can influence the depth of sequencing coverage and the possibility of further exploring mechanisms of resistance other than *MET* amplification. Gain and loss of gene copy numbers can be more difficult to confirm in plasma than in tissue. Nevertheless, this report provides valuable proof of concept for exploring the genomic mechanisms of resistance to *MET* inhibitors in the clinic.

In summary, this is the first report using NGS of ctDNA to monitor acquired resistance during anti-*MET* treatment in advanced GBC. Our data indicate that ctDNA can be a useful tool for tracking acquired therapeutic resistance and to analyze mechanisms of resistance. In the era of precision therapy, the accurate detection and identification of therapeutic targets should be prioritized in advanced BTC cases that have failed conventional treatments. This is a successful clinical case report of a GBC patient with *MET* amplification who received crizotinib as a monotherapy. Our findings suggest that crizotinib may be a promising therapeutic option for GBC patients with *MET* amplification and warrants further clinical investigation. In this study, the patient unavoidably became resistant to *MET* inhibitors and the mechanisms of resistance to *MET* inhibitors in patients with *MET* amplification remain unknown and require study.
